# Successful Determination of Larval Dispersal Distances and Subsequent Settlement for Long-Lived Pelagic Larvae

**DOI:** 10.1371/journal.pone.0032788

**Published:** 2012-03-12

**Authors:** Pelayo Salinas-de-León, Timothy Jones, James J. Bell

**Affiliations:** School of Biological Sciences, Victoria University of Wellington, Wellington, New Zealand; University of Hull, United Kingdom

## Abstract

Despite its importance, we still have a poor understanding of the level of connectivity between marine populations in most geographical locations. Taking advantage of the natural features of the southeast coast of New Zealand's North Island, we deployed a series of settlement stations and conducted plankton tows to capture recent settlers and planktonic larvae of the common intertidal gastropod *Austrolittorina cincta* (6–8 week larval period). Satellite image analysis and ground truthing surveys revealed the absence of suitable intertidal rocky shore habitat for *A. cincta* over a 100 km stretch of coastline between Kapiti Island to the south and Wanganui to the north. Fifteen settlement stations (3 replicates×5 sites), which were used to mimic intertidal habitat suitable for *A. cincta*, were deployed for two months around and north of Kapiti Island (at 0.5, 1, 5, 15, 50 km). In addition, we also conducted plankton tows at each settlement station when the stations were first deployed to collect *A. cincta* larvae in the water column. On collection, all newly settled gastropods and larvae in the plankton samples were individually isolated, and a species-specific microsatellite marker was used to positively identify *A. cincta* individuals. Most of the positively identified *A. cincta* settlers and larvae were collected at the first three sampling stations (<5 km). However, low numbers of *A. cincta* settlers and larvae were also recorded at the two more distant locations (15 and 50 km). Dispersal curves modeled from our data suggested that <1% of gastropod larvae would travel more than 100 km. While our data show that most larvae are retained close to their natal populations (<5 km), a small proportion of larvae are able to travel much larger geographic distances. Our estimates of larval dispersal and subsequent settlement are one of only a few for marine species with a long-lived larva.

## Introduction

Determining the scale at which marine larvae disperse and successfully settle remains poorly understood [1], [2]. Determining larval dispersal distances and subsequent successful settlement is important because the distance that larvae travel plays a critical role in founding new populations and maintaining existing populations [3]. Measuring the exchange of larvae between populations, or so-called ‘connectivity’ is critical for understanding how populations are linked via adult and larval stages (broadly termed population connectivity) and is especially relevant for the design of marine reserve networks, fisheries management and invasive species ecology [4].

Connectivity controls: 1) a population's buffering potential from local catastrophes and therefore its extinction risk [3]; 2) a population's potential as a source of new larvae to other populations [5]; and 3) the level of genetic mixing or differentiation between populations [6]. However, connectivity is not only a function of how far larvae travel, but it also depends on the number and frequency of larvae being exchanged between populations, the proportion of those individuals reaching new populations that reach maturity, and the influence of biological and physical processes that may act as barriers (e.g. distance and currents patterns) to prevent or enhance larval flow [7]. Connectivity can be further divided into demographic connectivity, which is the linkage between populations during the most recent generations whereby larvae settle and reach reproductive maturity, and genetic connectivity, which provides information on the genetic make-up of populations and how genes have been shared between populations, usually over a large number of generations (1000s) [8], [9]. Demographic connectivity is very difficult to measure compared to genetic connectivity, but is generally more relevant to environmental managers, as it provides information relevant to the typically short time-scales at which management operates. While there have been a considerable number of genetic connectivity studies across the world, there are very few world-wide estimates of the demographic connectivity of populations (but see [10]). Furthermore, the demographic connectivity estimates that are available are generally for species whose larvae only spend a short period of time in the water column (hours to days). This seriously inhibits our potential to model and understand both small- and large-scale connectivity patterns, and therefore effectively manage marine populations.

Direct measurements of larval dispersal distances and successful settlement in order to estimate demographic connectivity have been difficult to make due to the small size of most larvae and the comparatively large size of the oceans [11]. Furthermore, successful arrival at a location and settlement of a larva may not necessarily lead to the subsequent recruitment of the individual to the adult population or demographic connectivity. However, the first step to quantify demographic connectivity is to quantify the dispersal distances of larvae that result in successful settlement. While a number of larval dispersal distances have been estimated from collecting plankton or observing larval periods in aquaria, few studies have successfully measured either the subsequent settlement or recruitment in the field after a period of dispersal. This is because the larvae of most marine species can stay in the plankton for days to months and it is difficult to identify sources of settlers or recruits. Therefore in many cases larval transport and the associated transport processes have been inferred through other techniques, such as genetic markers and hydrodynamic models [12], rather than from direct measurements. The traditional view has been that larvae can be transported over large distances by oceanic currents and then settle; therefore marine populations were considered to be demographically ‘open’ [13]. However, recent research has shown that a large proportion of the larvae of many species may be retained close to their parental habitat and therefore marine populations might be less open than previously expected [7], [14], [15], [16].

Most previous estimates of larval dispersal distances have been inferred from the genetic characterisation of adult populations [17], [18], [19] or the use of natural or artificial markers to follow larval movement [20], [21]. However, most of the studies that have measured dispersal have been restricted to species with pelagic larval durations of only a few hours or days [22], [23], [24], [25]. Measuring the effective dispersal distance of species with long-lived larvae is much harder because of the dilution effect of larvae in the water column and the problem of identifying marine invertebrate larvae to species-level by morphological examination, as many diagnostic features are absent or poorly developed in early larval stages [26], [27]. Several authors have used genetic techniques to distinguish between morphologically similar larvae from plankton samples and newly established settlers [27], [28], [29]. Microsatellites are polymorphic loci that generally occur in the non-coding regions of nuclear DNA that consist of repeats of one to six base pairs in length [30]. Because microsatellites are usually species-specific markers, they can be used to identify newly established settlers or a larva of given species isolated from a plankton sample based on a single polymerase chain reaction (PCR).

In this study, we used a combination of settlement stations (mimicking rocky intertidal habitats in areas where there is no intertidal rocky habitat) and plankton sampling at the settlement stations to collect recent settlers and larvae at different distances from their most likely source population based on distance and information on water currents. A single microsatellite marker was then used to positively identify larvae of a common New Zealand intertidal gastropod in these samples to confirm the presence of a rocky shore species on our settlement stations and in the plankton samples. We then modeled dispersal kernals for our data to estimate the likely median distance where settlement would be less than one per station. This information provides a field-based estimate of how far larvae with a long (6 to 8 weeks) pelagic duration are able to actually travel and successfully settle.

## Results

### Larvae settlement to artificial islands

**Figure 1:**
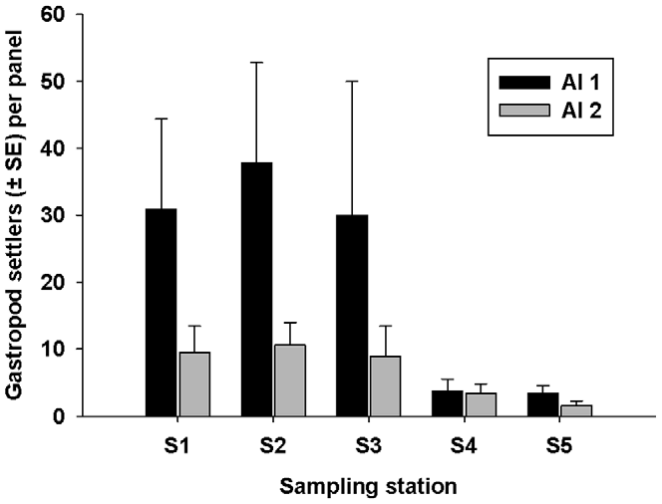
Mean number of gastropod settlers (± SE) per settlement panel (n = 8) found on artificial islands (n = 2) at different distances from Kapiti Island (S1–S5). S1 = 1 km S, S2 = 0.5 km N, S3 = 5 km N, S4 = 15 km N, S5 = 50 km N.

**Figure 2:**
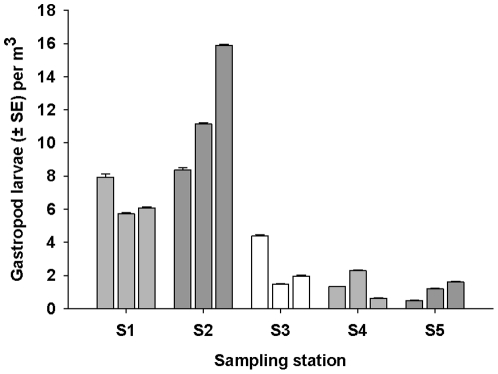
Mean number of *Austrolittorina cincta* settlers (± SE) per settlement panel (n = 8) found on artificial islands (n = 2) at different distances from Kapiti Island (S1–S5) identified using a microsatellite markers. S1 = 1 km S, S2 = 0.5 km N, S3 = 5 km N, S4 = 15 km N, S5 = 50 km N.

Of the 15 Artificial Islands (AIs) that we deployed, 13 were successfully recovered two months after deployment. In order to have a balanced design for the analysis, we used the same number of AIs per station, meaning that only data from 2 AIs from each station were included in the final analysis. In addition, some of the 16 panels on each of the AIs were missing; therefore only 8 panels (4 panels from the inside and 4 panels from the outside of the PVC pipe were haphazardly chosen from those remaining) per AI were examined to ensure the same numbers of panels were sampled across all stations. A total of 1088 gastropod settlers were recorded on the 80 settlement panels analysed (Fig. 1), and PERMANOVA identified a significant difference between stations, but not between AIs within a station (Table 1). Of the collected gastropod settlers, the highest proportion (91.4%; n = 995) was found at stations S1–S3, which were those closest stations to Kapiti Island (Fig. 1). Microsatellite analysis positively identified 433 (39.7%) of the 1088 gastropod settlers as *A. cincta* settlers, and *A. cincta* settlers were positively identified at all of sampling stations although most (90.5%; n = 392) were recorded from the three stations closest to Kapiti Island (Fig. 2). PERMANOVA indicated a significant difference between stations, but not between AIs within each station (Table 2).

**Table 1:** Permutational analysis of variance (PERMANOVA) based on a Bray-Curtis similarity matrix, testing the effect of station (5 levels fixed) and artificial island (2 levels, random) on the total gastropod abundance on settlement panels.

Source	DF	SS	MS	Pseudo-F	P(perm)	Permutations
Station (s)	4	27908	6977	3.7	0.08	623
AI (ST)	5	9474	1895	1.3	0.20	999
Res	70	1.019×10^5^	1456			
Total	79	1.3928×10^5^				

Statistical differences were tested using 9999 permutations under a reduced model.

**Table 2:** Permutational analysis of variance (PERMANOVA) based on a Bray-Curtis similarity matrix, testing the effect of station (5 levels fixed) and artificial island (2 levels, random) on the abundance of *A. cincta* on settlement panels.

Source	DF	SS	MS	Pseudo-F	P(perm)	Permutations
Station (s)	4	27447	6862	6.04	0.001	997
AI (ST)	5	6363	1272	1.12	0.33	996
Res	70	79480	1135			
Total	79	1.13×10^5^				

Statistical differences were tested using 9999 permutations under a reduced model.

### Plankton tows

**Figure 3:**
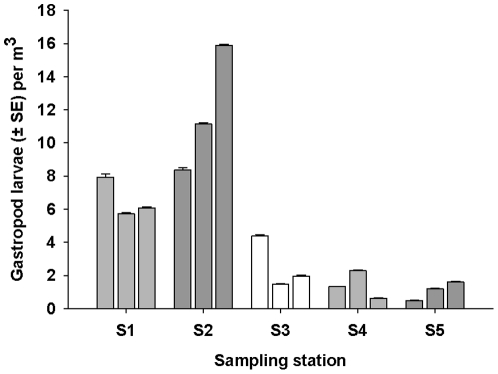
Mean number of gastropod larvae (± SE) per m^3^ of filtered seawater (n = 9) found at different distances from Kapiti Island (S1–S5). S1 = 1 km S, S2 = 0.5 km N, S3 = 5 km N, S4 = 15 km N, S5 = 50 km N.

A total of 15 plankton tows were conducted at the 5 different sampling stations and 1011 gastropod larvae were recorded in the 300 ml subsample (150×2 ml subsamples) (Fig. 3). Forty percent of the 15 plankton tows were sub-sampled (due to the large number of larvae they contained), and the total number of gastropod larvae present was 2527 individuals with a density of 4.69 individuals per m^3^ of seawater sampled. PERMANOVA identified a significant difference between the abundance of gastropod larvae between stations (but not between samples within stations), and larvae were more abundant at the stations closer to Kapiti Island than those further away (Fig. 3).

Of the total 1011 gastropod larvae recorded in the plankton tows, 247 (50 were haphazardly selected per station across all AIs, except station 5 where all 47 larvae collected were analysed) were assayed using the microsatellite markers. *A. cincta* larvae were positively identified at all sampling stations with a total of 18 larvae out of the 247 (7.2%) being positively identified as *A. cincta* larvae. Eight percent of the larvae at Station 1 were positively identified as *A. cincta* larvae; 12% at Station 2: 8% at Station 3: 4% at Station 4; and 4% at Station 5. As this was a subsample of the entire plankton sample, extrapolation of this data provided an estimate of 242 *A. cinta* larvae in all of the plankton samples, with a total overall concentration of 2.6 *A. cinta* larvae per m^3^ of water.

### Modeling dispersal curves

**Figure 4:**
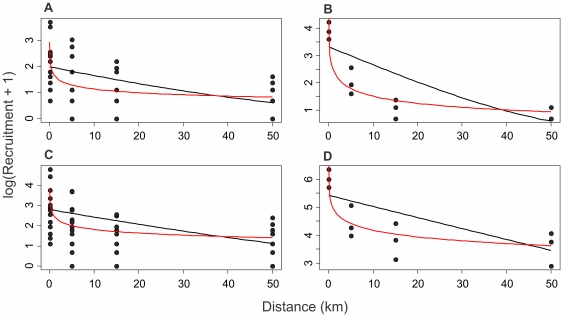
Recruitment (Log) plotted as a function of distance for *A. cincta* recruits (a), planktonic *A. cincta* collected in plankton samples (b), all gastropod recruits (c) and all planktonic gastropods collected in plankton samples (d). Lines illustrate fitted models for exponential models (black lines) and power law models (red lines).

The resulting model parameters are given in Table 3. Based on AIC, negative binomial models were a better fit to the data (lower AIC values) and therefore the final analysis was based on negative binomial fits to the data. For all datasets the power law model was a better fit to the data (ΔAIC 5 to 18 indicates weak to very weak support for the exponential model over the power law model, [31]). Power law parameters for *A. cincta* recruits, all gastropod recruits, and all gastropod larvae were in the range of −0.31 to −0.35 (95% CI −0.2 to −0.45) indicating a consistent reduction in recruits and all gastropod larvae with distance from the most likely source. The counts of *A. cincta* planktonic larvae showed a sharp decline with distance indicated by a more negative power law parameter (*γ* = −0.5, 95% CI −0.59 to −0.41), however, due to the overlap of 95% confidence intervals for power law parameters they are not significantly different at the 5% significance level. When the fitted models were plotted over the data, both models appear to model the decline in recruits with distance (Fig. 4). However, the exponential model tended to overestimate the number of recruits for *D* = 5 km and *D* = 15 km and underestimate the number of recruits for *D* = 0.05 km. In contrast, the power law model provided an accurate fit to the data, which was particularly evident when log(*R*) is plotted against log(*D*) (data not shown), and showed no serial bias in the model fit to the data. Distance estimates *D_1_* and *D_0.5_* from the power law models were highly variable, with large 95% confidence intervals. This is a result of the power law relationship having a strong negative slope at small distances but then as *D* increases the slope becomes much flatter and so would take greater distances for *R* to reach lower values. However, these distances were fairly consistent between *A. cincta* recruits and *A. cincta* larvae with median dispersal distances of *D_1_* = 113, 133 km and *D_0.5_* = 1046, 523 km, respectively.

**Table 3:** Model parameters plus 95% confidence intervals for the distance.

Dataset	Model	*R_0_/R_1_*	*β*	*γ*	*D_1_* (km)	*D_0.5_* (km)	AIC
All settlers	Exponential	15.5(10.9, 23.1)	−0.04(−0.06, −0.03)	/	68(52, 103)	84(65,131)	402
	Power law	10.8(8, 15.2)	/	−0.32(−0.44, −0.21)	1816(272, 8.7×10^4^)	1.6×10^4^(1.3×10^3^,2.5×10^6^)	396*
*A. cincta* settlers	Exponential	6.3(4.2, 9.8)	−0.04(−0.06, −0.02)	/	44(32,73)	61(45,102)	297
	Power law	4.3(3.1, 6.3)	/	−0.31(−0.44, −0.19)	113(27, 2445)	1046(145, 9.3×10^4^)	292*
All plankton gastrop	Exponential	226(138, 403)	−0.04(−0.06, −0.02)	/	139(95,347)	156(106,396)	141
	Power law	148(114, 198)	/	−0.35(−0.45, −0.26)	1.3×10^6^(5×10^4^,6.5×10^8^)	9.4×10^6^(2×10^5^, 8.4×10^9^)	128*
*A. cincta* plankton	Exponential	26.9(13.6, 60.5)	−0.07(−0.11, −0.04)	/	46(31,87)	56(38,106)	82
	Power law	11.7(9.0, 15.2)	/	−0.50(−0.59, −0.41)	133(42,579)	523(132,3200)	64*

All parameter values are maximum likelihood estimates except *D_1_* and *D_0.5_* which were the median estimates for these distances obtained from OpenBUGS. All models were fit assuming negative binomial errors. * indicates the better fit of the two models.

## Discussion

Our results indicate that a high proportion of the long-lived pelagic larvae of *A. cincta* settled within a 5 km range of the likely source population, but that a small proportion of larvae were able to travel and successfully settle as far as 50 km away. Furthermore, this movement of larvae appears to be in the opposite direction to the predominant water currents of the region. Our dispersion kernals suggested that <1% of gastropod larvae would travel more than 100 km. It is important to note that the actual decline in the number of larvae and settlers is likely to be the result of a dilution effect of larvae, and not necessarily a decrease in the number of larvae reaching the more distant stations. Our data represent one of the few direct estimates of larval dispersal distance followed by successful settlement for a species with a relatively long-lived larval stage. While these estimates may not represent demographic connectivity (since the snails had not reached reproductive maturity by the time we collected them), the fact that the larvae had settled and developed into juvenile snails means this is the likely distances over which demographic connectivity occurs for *A. cincta*.

There has been much debate about the ‘closed’ versus ‘open’ nature of marine populations, and our data suggest that a high level of larval exchange and subsequent settlement occurs between populations close-by (<5 km) compared to more distant populations, but also provides evidence that larvae are able to travel much greater distances, but with reduced frequency. Therefore, the terms ‘open’ and ‘closed’ may be uninformative for many species as it appears that for any given species with a pelagic larva, there will be a wide range of potential and achieved dispersal distances along a spatial continuum; some larvae will be effectively self-recruited, while others will be dispersed more widely [9], [14]. However, while our results demonstrate that a large proportion of larvae settle close to the most likely adult population, in other areas where strong currents prevail, it is possible that a greater proportion of larvae are advected away to more distant locations [10], [13], [16].

Preliminary observations of *A. cincta* larvae in the laboratory suggest they are not strong swimmers, but are likely to be capable of vertical movements, so we propose that the distances travelled in this study are likely to be influenced by the oceanographic features of this part of New Zealand. The west coast of New Zealand's north Island is under the influence of three major current systems: the West Auckland current flowing south from the top of the NZ north island; the D'Urville current flowing south-east through Cook Strait and the Westland current flowing north from the west of the south Island [32]. Of these currents, the D'Urville current is thought to be responsible for the predominant flow around Kapiti Island. Chiswell and Stevens (2010) used Lagrangian and Eulerian measures to estimate current flow around Kapiti Island and reported that the mean flow was to the south-west, towards the Cook Strait [33]. It is therefore surprising that we found *A. cincta* at our settlement stations north of Kapiti Island, the most likely source of larvae based on distance. Finding larvae 50 km north of Kapiti Island suggests that larvae either moved against prevailing southward water currents or had drifted to that station from the north. In either case, drift distances were about 50 km. However, despite the previous modeling of the water flow in the vicinity of our study area (Kapiti Island), more recent and ongoing studies suggest that although the predominant current direction is in a southerly direction toward Cook Strait, in periods of sustained southerly wind, a wind-induced north-flowing current can be established [34]. We believe that this wind-induced flow reversal facilitated the northward movement of larvae from Kapiti Island and its southern coast, as the period when our study was conducted contained long periods of southerly wind flow [34].

An alterative explanation for the occurrence of *A. cincta* settlers on our stations or for the larvae in our plankton samples is that they originated from the northern Wanganui populations. While we are not able to completely exclude this possibility, particularly at the most distant station from Kapiti Island, we believe this to be unlikely for the stations closer to Kapiti Island given the decline we observed in overall gastropod settlers/larvae and also *A. cincta* settlers/larvae with increasing distance from Kapiti Island. If the northern population were the source, then the *A. cincta* larvae would still have been dispersing a minimum distance of 50 km (as S5 was located halfway between Kapiti Island and Wanganui), and potentially dispersing up to 75 or 95 km if they had managed to reach S4 and S3, respectively. While it possible that some of the settlers and planktonic larvae collected at the sites closest to Kapiti Island originated further north, although given all the other evidence above this number is likely to be only a small fraction of the total number of settlers/planktonic larvae. A previous genetic analysis of adult *A. cincta* populations using a suite of microsatellite loci [35] found no genetic differentiation between Kapiti MR and Wanganui populations (F_ST_<0.001, P>0.05), suggesting that some larvae must travel the 100 km that separate these two populations (given there is no suitable habitat in between) to account for the homogenous genetic structure; this is also supported by our dispersal kernals that suggest the median dispersal distance where settlement reaches less than one is around 100 km. Furthermore, the lack of genetic structure between these locations (coupled with Baysian analyses [35]) means larvae are travelling in both a north and southward direction. This previous genetic study also showed that significant genetic differentiation for this species can occur at spatial scales less than <50 km, which coupled with the data from the present study suggests that maximum dispersal distance and likely range of demographic connectivity of this species is in the range of 50 to 100 km. While we believe that the most likely sources of the larvae found on our panels is from the south, given the similarity of settler numbers at S1 (which was deployed in continuous adult *A. cincta* habitat) and S2/S3, it is possible that they could have originated from anywhere along the southern rocky coastline, and we cannot exclude the possibility that settlers found on the panels travelled a considerable distance from more southerly locations. However, once again the rapid decline in larvae with increasing distance from the last source of rocky shore (for *A. cincta* and all larvae) strongly suggests that most of the larvae originate from the rocky shore close to Kapiti Island.

There are relatively few dispersal estimates for species with a long pelagic larval duration (e.g. weeks). McQuaid and Phillips [36] determined the dispersal potential of the invasive intertidal mussel *Mytilus galloprovincialis* in South Africa by investigating larval movement from a single point source. These authors revealed that the maximum effective dispersal distance of mussel larvae in that area was within the scale of <100 km, with the great majority (90%) of individuals remaining just 5 km from the point of origin four years after the invasion; this figure is similar to our study, given that we recorded 90% of *A. cincta* settlers within 5 km of Kapiti Island. Similarly, Becker et al. [21] used elemental fingerprinting to estimate mussel larval movement on the Californian coast. These authors found that mussel larvae can be retained within 30–35 km of their natal origin, a dispersal figure also similar to our study. It is difficult to compare our estimates of larval movement to most previous studies as the majority have been conducted on species with a very short pelagic larval duration (e.g. [23], [37]. However, Dethier et al. (2003) found the abundance of several benthic species were recorded on an artificial exposed rocky jetty surrounded by dissimilar habitat (i.e. sandy beaches) on the coast of Washington state (USA), to those at the two closest rocky shore sites (43 km to the north and 70 km to the south) [38]. These authors found the littorinid gastropods that had a planktonic larval dispersal stage (*Littorina scutulata* and *Littorana plana*) living on the jetty and although they did not directly measure the dispersal distance for these gastropod species, they suggested that the larvae must have dispersed at least 43 km, as this was the distance to the nearest larval source. Planes et al. [10] used DNA parentage analysis on the orange clown fish *Amphiprion percula*, whose pelagic larva spends approximately 11 days in the water column. These authors reported the longest direct measure of larval dispersal for any marine fish species to date, given that they located juveniles that were produced by adults on the isolated Kimbe Island on neighboring reefs up to 35 km away; a dispersal distance similar to the one obtained in our study. These past studies, combined with our data, suggest that larval dispersal distances for species with pelagic larval durations of weeks are typically likely to be in the range on 5–100 km, although the level of population demographic connectivity will decrease with increasing distance between populations as dilution effects take place.

## Materials and Methods

**Figure 5:**
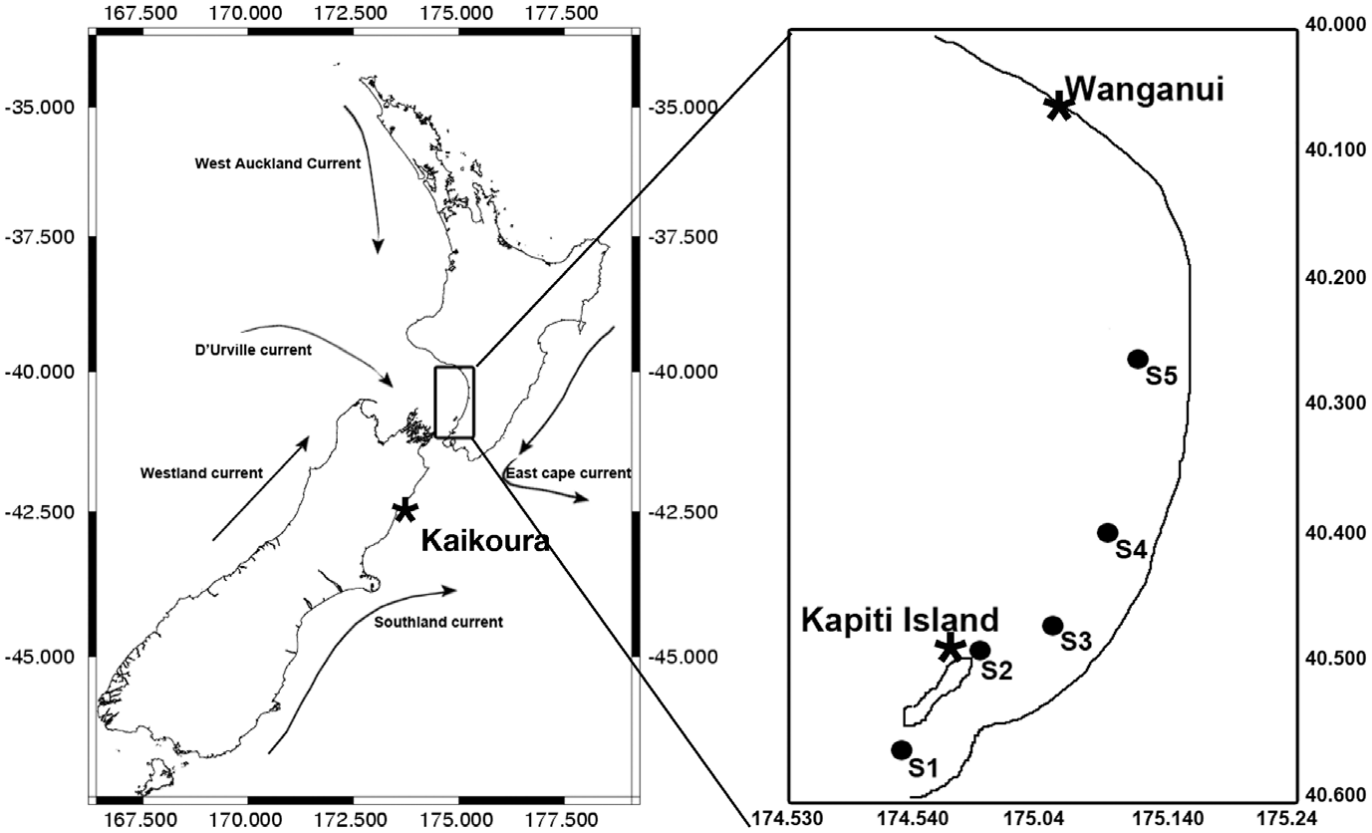
Map showing coast where artificial islands were deployed.

This study was designed to take advantage of the natural features of the Kapiti-Wanganui coast on the southwest coast of the New Zealand's North Island (Fig. 5). Satellite image analysis corroborated with ground truthing surveys revealed the absence of suitable intertidal rocky shore habitat from Kapiti Island (KI) (S40°49′13″; E174°56′31″) and the coast in front of KI, all the way to Wanganui jetty (WA) (S39°56′55″; E174°58′47″) located over 100 km to the north. This lack of suitable intertidal habitat meant that KI to the south (and the coast southwards) and WA to the North (and all the coast northwards) were the only sources of larvae for this species along this 100 km stretch of coastline. The mean water flow direction around Kapiti Island, as revealed by Lagrangian and Eulerian estimates, is to the south-west, towards the Cook Strait [33]. However, more recent local-scale studies have shown that wind-driven flow patterns cause water flow to the north during periods of sustained southerly winds [34]. *Austrolittorina cincta* was selected as the main focus for this study given the availability of microsatellite markers for this species together with its relatively well-known ecology [39], [40]. The availability of microsatellite markers enabled us to distinguish *A. cincta* larvae from all other mollusc larvae (which are not distinguishable by eye) in mixed samples. *A. cincta* is a small intertidal gastropod (generally <10 mm) that is commonly found on the upper shore of rocky habitats throughout New Zealand. Vander-Veur [39] studied the ecology of this species on the New Zealand North Island and revealed very high population abundance (1600 individuals/m^2^), relatively low adult movement rates (18.7 m±16 m/year) and that settlement peaked between February and March. Mating occurs in December and January, and the veliger larvae spend approximately 4–6 weeks in the water column before settling to the lower shore and then migrating to their higher shore adult habitat.

### Settlement to Artificial Islands

Five sampling stations were selected at different distances from KI and each station consisted of three replicated artificial islands (AIs; n = 15). Station 1 was located 1 km south of KI (to estimate settlement rates in areas of continuous *A. cincta* habitat); station 2 was located 0.5 km north of KI; station 3 was located 5 km north of KI; station 4 was located 15 km north of KI; and station 5 was located 50 km north of KI (Fig. 5). Stations were marked with a GPS location for ease of relocation. As a result of logistical constraints it was not possible to conduct the experiment all the way from KI to WA (i.e. the full 100 km).

**Figure 6:**
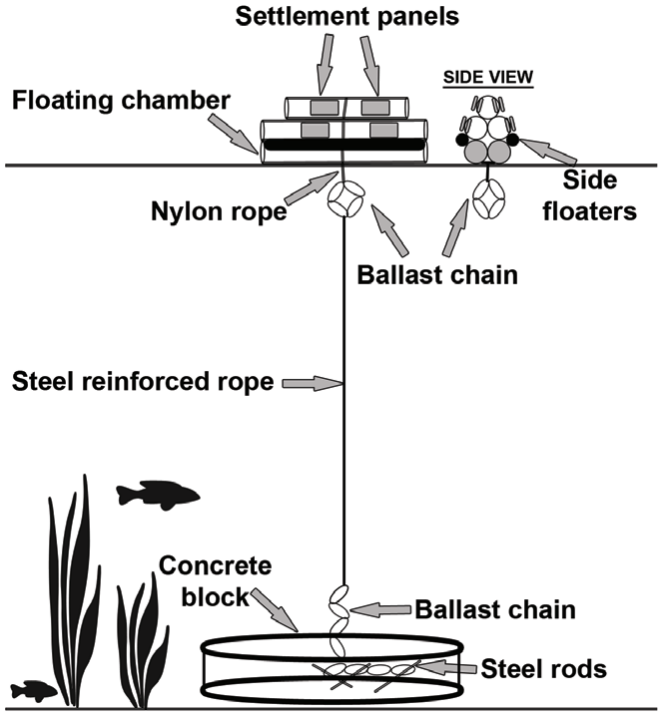
Schematic representation of an artificial island.

**Figure 7:**
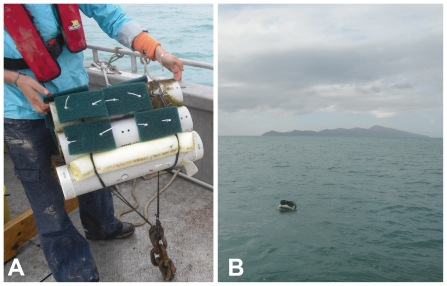
Experimental setup. (a) Artificial Island (AI) ready for deployment (b) Deployed AI near Kapiti Island.

AIs were designed to provide artificial intertidal habitat for the intertidal gastropod *A. cincta* larvae to settle on and were based on a preliminary intertidal study, where *A. cincta* were readily found to settle on artificial Scotch-Brite© scouring pads [39]. Each AI was moored to a concrete anchor and deployed at around 15 m depth on the sandy seabed to try to minimise the impact of swells on station movement (Figs. 6 and 7). A 3 m chain was attached to each mooring to serve as extra anchor weight; 20 cm of the chain was embedded in the concrete and three pieces of steel reinforced rods 30 cm in length were passed through 3 different chain links before the concrete was poured to avoid the chain being detached from the concrete mooring. A 40 m steel reinforced rope connected the mooring chain to the AI. Each AI consisted of 5 pieces of PVC pipe 60 cm in length: 2 pieces were sealed with PVC pipe end caps and were glued together to create an air filled flotation chamber, while the remaining three PVC pieces were glued together in a triangular shape and then glued to the air filled pieces. Two polystyrene floaters were attached to the side of the AI to provide extra buoyancy and help stabilise the AI. Five meters of nylon rope was strapped around the entire AI to ensure all the glued PVC pieces stuck together and also to attach the ballast to the AI. An initial experimental trial revealed that 2.5 kg of chain ballast was adequate to ensure the AI would return to its original floating position if it was tipped over by waves. The AIs were influenced by splash to mimic intertidal habitats. A metal clip was used to join the steel reinforced rope from the mooring to the ballast chain of the AI.

Sixteen settlement panels were placed on each AIs: 8 on the inside of the PVC pipes and 8 on the outside of the PVC pipes (Figs. 6 and 7). Settlement panels were 15×8 cm Scotch-Brite© scouring pads (kindly donated by 3 M New Zealand). Previous pilot studies [37] revealed that scouring pads were the most suitable substrate to study *Austrolittorinid* settlement. Sets of holes were drilled through the top three PVC pipes and through the settlement panels, and panels were attached by means of cable ties through the panels and PVC pipes holes (Figs. 6 and 7).

AIs were deployed for a two-month period between 1^st^ February and 30^th^ March 2009. This time for deployment was based on a previous recruitment study that revealed that *A. cincta* settle as this time of the year [39]. On collection, panels were labeled, preserved in 95% ethanol and transported to the laboratory. Each panel was cut into 4 pieces, and each piece was flushed with running water and all settlers present on each panel were collected on a 100 µm mesh sieve located below. The biological material collected in the sieve mostly comprised intact specimens (>95%), demonstrating the removal method destroyed very few larvae. The contents on the sieve were re-suspended in 50 ml of 95% ethanol for subsequent microscope analysis. All the gastropod and bivalve larvae present in each of the samples were recorded using a dissecting microscope on a Bogoroff tray. Individual gastropod larvae were collected using a micropipette and placed in individual 1.5 ml Eppendorf tubes for later molecular analysis.

### Plankton tows

In addition to the settlement study, plankton tows were conducted to quantify the abundance of *A. cincta* larvae in the water column at the time when the AIs were deployed. The plankton sampling (1^st^ February) coincided with the known settlement period of *A. cinta* (see above). While this represents only a snapshot of larval availability compared to data from our settlement stations, the high abundance of larvae at this time interval enabled us to estimate if any larval gradients existed. Three replicate plankton tows were collected at each of the five sampling stations (n = 15). We used a plankton net with a 50 cm diameter and constructed of 100 µm mesh. The net was towed behind the boat near the water surface (<0.5 m) at a constant speed of 3 knots in a straight line. The GPS locations at the start and finish of the tow were also recorded and compared to the estimates of distance moved calculated below (in all cases they were within 5%). Trial plankton tows for >3 minutes showed evidence of net clogging at the site closest to Kapiti, therefore the net was towed for 2 minutes and the total length of each tow was calculated as:

3 knots = 5.55 km/h; 5.55 km/h = 1.53 m/s; 1.53 m/s×120 s = 183 m.

Given the 50 cm diameter of the plankton net, the area of the net opening is:

A = π×r^2^; A = 3.14×(0.25 m)^2^; A = 0.19 m^2^.

Assuming a 100% net efficiency, each plankton tow filtered:

Total water filtered = Length of tow×Net area; T = 183 m×0.19 m^2^; T = 35.9 m^3^.

The plankton sample was transferred to a 500 ml collecting vessel using an opening fitted at the cod end of the net, preserved in 95% ethanol and transported to the laboratory for posterior analysis. Plankton samples were analysed using a dissecting microscope to quantify all the gastropod and bivalve larvae present. Given the high abundance of organisms, 5×2 ml sub-samples (sampled using a plankton splitter) were analysed from each tow in a Bogoroff tray. Individual gastropod larvae were collected using a micropipette and placed in individual 1.5 ml Eppendorf tubes for subsequent molecular analysis.

### Molecular analysis

The DNA from individual isolated gastropod larvae and settlers was extracted using a Chelex extraction method. 50 ul of Chelex (BioRad) 20% was added to each tube containing a larva, incubated for 60 min at 55°C, followed by 10 min at 95°C and then centrifuged at maximum speed for 3 min. We used the species-specific microsatellite, D104 [40] to confirm the presence of *A. cincta*, because this locus produced consistent product and did not cross amplify with the closely-related sister species *A. antipodum* (which is also a common intertidal species in the locality).

Polymerase chain reactions (PCR) were conducted on a MJ Research thermocycler in 15 ul reaction volumes containing 2 ul 10× buffer (Roche) including 1.5 mM MgCl_2_, 1 ul 10 µM dNTPs, 1 ul of 10 µM of each primer, 0.1 ul *Taq* DNA polymerase (Roche) and 20 ng of template DNA. Amplifications were conducted under the following conditions: 95°C for 5 minutes, followed by 40 cycles at 95°C for 30 seconds, 54°C for 30 seconds, 72°C for one minute, and a final extension step ay 72°C for 30 minutes. The presence of PCR products was confirmed on a 3% agarose gel and on each PCR cycle a positive control (i.e. using previously genotyped *A. cincta* adult DNA) and a negative control was (no DNA) included to avoid false positives.

### Data analysis

Differences in the total number of gastropod settlers and the number of *A. cincta* on the panels, and total larvae and *A. cincta* larvae in the plankton tows at the different stations were analysed by the statistical package PRIMER v6 and PERMANOVA v1.0.2. PERMANOVA is a permutation-based multivariate analysis of variance [41], which uses the distances between samples to partition variance and randomisations or permutations of the data to produce the p-value for the hypothesis to be tested. It is non-parametric and, therefore, robust to the assumption of normality making it less prone to Type I errors. The PERMANOVA model included one factor (sampling station) for both the settlers and larvae datasets.

### Modeling dispersal curves

Counts of planktonic larvae and recruits were modeled as a function of distance to assess the maximum dispersal and recruitment distances for *A. cincta* and for all gastropods. As the response variable was counts of individuals the response is likely to be poisson distributed [42]. However, ecological data are often overdispersed compared to a poisson distribution (this would particularly be expected for counts of all gastropods as we have a mix of species that may have different larval dispersal abilities and different abundances giving rise to a mix of counts) and is better modeled by a distribution that can account for this overdispersion, such as the negative binomial distribution [42]. We considered two functional forms to model the number of recruits and planktonic larvae as a function of distance. The first was that of an exponential decline in abundance *R* with distance *D* given by
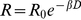
(1)Where *R_0_* is the number of recruits/larvae at distance *D* = 0 and *β* is a parameter estimating the rate of decline of recruits/larvae with distance. This was modeled using log linear models with both poisson and negative binomial errors distribution models (glm and glm.nb with log link in R version 2.13.2, R development core team 2011) to estimate the parameters and 95% confidence intervals for *R_0_* and *β*. The second function was a power law with exponent γ controlling the rate of decline of recruits/larvae with distance
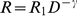
(2)where *R_1_* is the number of recruits/planktonic larvae at a distance *D* = 1. This was modeled using log-linear models but with *log(D)* as the predictor as taking the logarithm of equation 2 we get an equation which is linear in *log(D)*

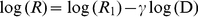
(3)This was modeled using both poisson and negative binomial errors distribution models (glm and glm.nb with log link in R version 2.13.2 [43]) to estimate *R_1_* and *γ* plus 95% confidence intervals. Models were then compared based on AIC value to identify the most appropriate fit to the data.

Maximum dispersal distances were estimated by setting *R* = 1 and *R* = 0.5 in equations 1 and 2 to indicate distances beyond which you would expect to see less than 1 (*D_1_*) or less than an average of 0.5 (*D_0.5_*) individuals settling or as planktonic larvae. The distances plus 95% confidence intervals were then estimated using a Markov Chain Monte Carlo method in OpenBUGS [43]. Although OpenBUGS is most often used for Bayesian analysis, one can choose to perform a strictly likelihood based analysis by specifying that the prior distributions for estimated parameters are uniform and in which case the posterior distributions are simply the same as the likelihood distribution (known as an empirical Bayesian analysis, [43]). Models run in OpenBUGS were used to solve for the model parameters (*R_0_*, *β* or *R_1_, γ* depending on the model) using the same model specifications as above. Once the model had reached a stable solution (models were run with three chains to identify model convergence) we obtained model parameters from the model solution and then used these values to solve for distances *D_1_* and *D_0.5_*. Chains were run for a total of 30000 samples with each sample obtained every 5 iterations to reduce the impact on parameter estimates of correlations in model parameters between iterations. We discarded the first 10000 samples as a burn in period and used the remaining 60000 samples (20000 from each of three chains) to estimate the median and the 95% confidence interval for distances *D_1_* and *D_0.5_*. The median was more representative of the average dispersal distance than the mean as large distance estimates were produced infrequently from the model fit, which resulted in heavily skewed mean distance estimates.
